# Infection with *Helicobacter pylori* and the age at onset of coeliac disease in the “HUNT4” survey

**DOI:** 10.1186/s13104-025-07594-5

**Published:** 2025-12-06

**Authors:** Kjetil K. Melby, Rebecka Hjort, Knut E. A. Lundin, Eivind Ness-Jensen

**Affiliations:** 1 Department of Microbiology, Oslo University Hospital, N-0407 Oslo, Norway; 2 Department of Microbiology, Faculty of Medicine, University of Oslo, Oslo, Norway; 3 HUNT Research Centre, Department of Public Health and Nursing, NTNU, Norwegian University of Science and Technology, Levanger, Norway; 4 HUNT Center for Molecular and Clinical Epidemiology, Department of Public Health and Nursing, NTNU, Norwegian University of Science and Technology, Trondheim, Norway; 5 Department of Medicine, Levanger Hospital, Nord-Trøndelag Hospital Trust, Levanger, Norway; 6 Norwegian Coeliac Disease Research Centre, Faculty of Medicine, University of Oslo, Oslo, Norway; 7 Department of Gastroenterology, Oslo University Hospital Rikshospitalet, Oslo, Norway; 8 Department of Molecular Medicine and Surgery, Karolinska Institutet and Karolinska University Hospital, Stockholm, Sweden

**Keywords:** Helicobacter pylori, Coeliac disease, Age at onset, H.pylori antigens, HUNT4 survey

## Abstract

**Objective:**

This study aimed to determine whether *H. pylori* or its antigens affect the age at which coeliac disease (CeD) was diagnosed. Participants over 20 years old from the HUNT4 survey were screened for CeD by measuring serum immunoglobulin A and immunoglobulin G antibodies against transglutaminase-2. *H. pylori* status was defined by detecting *H. pylori*-specific immunoglobulin G.

**Results:**

*H. pylori* + participants had a mean age of 62.3 years, compared to 54.8 years for negative participants. In those with previously undiagnosed CeD (*n* = 43), higher antibody levels against GroEL were associated with an older age at sampling (67.2 years for GroEL-positive vs. 50.8 years for negative). Smaller age differences were noted for gGT (6.0 years), UreA (8.6 years), and HpcC (6.4 years), while vacA and cagA showed only minor differences. Participants with CeD and *H. pylori* + were older than those who were *H. pylori*−. The presence of antigens such as GroEL, gGT, and UreA appeared to be associated with this age difference. Aside from *H. pylori* infection in childhood, a cohort effect of *H. pylori* infection may partly explain the differences.

## Introduction

Coeliac disease (CeD) is caused by an immune reaction to gluten, a protein in foods containing wheat, barley, or rye. Eating gluten triggers an immune response to the gluten protein in the small intestine. Over time, this reaction damages the small intestine’s lining and prevents it from absorbing nutrients, resulting in malabsorption. The intestinal damage often causes symptoms such as diarrhoea, fatigue, weight loss, bloating, and anaemia. It can lead to serious complications if untreated [1, 2]. There is no definite cure for CeD [1, 2]. For most people, however, following a strict gluten-free diet can help manage symptoms and support the healing of the intestines [1–4]. CeD generally occurs in genetically predisposed individuals, with either tissue type HLA DQ2 or HLA DQ8 [1–4]. These genetic markers typically occur in 30–40% of humans, although only about 2–3% develop the disease in their lifetime [1]. While many individuals with CeD develop the condition before the age of 10 years, it can also manifest later in life [1].

General laboratory manuals [5] describe various methods for detecting *H. pylori.* For large-scale epidemiological studies, the detection of specific *H. pylori* antibodies (typically IgG) may be suitable for identifying individuals with prior exposure.

Additionally, interactions between *H. pylori* and CeD have been documented [2–4]. As reports have indicated that *H. pylori* infection is inversely associated with allergic and autoimmune diseases [6–8], we previously conducted a small study that suggested that carrying *H. pylori* cagA+(cytotoxin-associated antigen A) strains may be linked to a delayed onset of CeD [9]. Thus, individuals harbouring *H. pylori* cagA + strains were older than those who were *H. pylori* negative [9]. The present study aimed to determine whether *H. pylori* or its antigens affected the age at which CeD was diagnosed.

## Materials and methods

Cases were obtained from the population-based HUNT4 survey in which all participants over 20 years of age were screened for possible CeD by measuring serum immunoglobulin A (IgA) and immunoglobulin G (IgG) antibodies against transglutaminase-2, as previously described [2–4]. Participants who tested positive for these serum markers of CeD (*n* = 1,107, denoted sCeD), of whom 53.1% were women, were invited to undergo an endoscopy. This procedure included histopathological examination of biopsies from the duodenum and testing for *H. pylori* infection in the stomach using a specific polymerase chain reaction (PCR). Of the 657 cases, only 48 were *H. pylori* positive.

In the present study, *H. pylori* infection was initially determined using IgG *H. pylori* serology (LIAISON^®^ *H. pylori* IgG Diasorin, Italy). The presence of *H. pylori*-specific IgG antibodies indicates presence of *H. pylori* if not given antibiotic treatment directed against *H. pylori* [5]. Subsequently, SeraSpot^®^ Anti-Helicobacter-6 IgG (Seramun Diagnostica GmbH, Germany) was applied for the detection of specific IgG antibodies against *H. pylori* antigens: cagA(cytotoxin-associated antigen A), gGT (a highly conserved virulence factor present in all Hp strains), GroEL (chaperonin – Heat shock protein 60), HpcC (Helicobacter cysteine-rich protein C), UreA (urease subunit A - a virulence factor), and vacA (vacuolising toxin). Only sera that provided a positive or borderline result in the primary test were subject to further analysis. In this specific antigen assay, six highly immunogenic *H. pylori* antigens were applied. Antigens in SeraSpot^®^ Anti-Helicobacter-6 IgG/IgA are based on recombinant proteins. These were expressed in and purified from *Escherichia coli* and spot-immobilised in wells of microtiter plates to detect serological immune responses in patient sera. Some strains produce toxins such as VacA and CagA [5, 10–14]. Studies have identified HcpC and GroEL as virulence factors, with GroEL potentially being more important than HcpC [11]. For the development of the spot immunoassay, six different *H. pylori* proteins were considered: CagA (P80200), VacA (Q48247), GroEL (P42383), UreA (P14916), gGT (O25743), and HcpC (O25728). The selection was based on antigen properties such as virulence factors (CagA, VacA, GroEL, UreA), immune evasion factors (gGT and VacA) [5, 10–14], and surface-localised or secreted *H. pylori* proteins (HcpC) epidemiologically linked to gastric cancer [11]. The tests for specific IgG antibodies in this test system were run according to the instruction manual provided by the producer [15].

## Results

Of the 1,107 individuals with positive sCeD, 958 participated in this study: 526 women (mean age 54.8 years) and 432 men (mean age 56.8 years). Of these, 952 had sera for anti-Hp-IgG testing, with 131 positives for IgG against *H. pylori*, 49 borderline, and 772 negatives. Results are given in Tables 1 and 2.

**Table 1:** Sera from the HUNT4 survey were tested for the presence of IgG anti-*H. pylori *antibodies

Relation to CeD (HUNT4)				
*H. pylori* serology	UCeD	KnCeD	Unclassified	Total
Female	354	36	198	588
Borderline	7	3	11	21
Insufficient volume	2	–	–	2
Negative	262	31	144	437
Positive	24	2	40	66
No serum	59	–	3	62
Male	311	23	185	519
Borderline	14	1	13	28
Insufficient volume	2	–	2	4
Negative	194	13	128	335
Positive	19	8	38	65
No serum	82	1	4	87
Total	665	59	383	1107

The samples were further categorised based on the information available at the time of serum collection into the following groups: UCeD: Unknown CeD; KnCeD: Known CeD; Unclassified (i.e. neither UCeD nor KnCeD): no classification available. All serum samples exhibited elevated antibody levels consistent with coeliac disease (sCeD), as detailed in the materials and methods section

**Table 2:** Mean age of participants with previously unrecognised coeliac disease (sCeD) (*n* = 520, of which data from 499 were available), based on the criteria given in materials and Methods

Status			Total
	Recently diagnosed CeD by serology (sCeD)		
	Hp negative	Hp positive	
Mean age	52.1	58.2	
No. of cases	456	43	499

Participants were further categorised by *H. pylori* (Hp) serology as carriers (positive) or non-carriers (negative)

Excluding known CeD (*n* = 64), non-CeD (*n* = 322), and lack of biopsy (*n* = 7) in individuals who were sCeD positive, a provisional potential or definite CeD diagnosis was reached in 559 cases, of which 351 had confirmed CeD. Of these, 26 (7.4%) were *H. pylori* positive and 13 (3.7%) had borderline results.

Individuals with sCeD who tested negative for *H. pylori* had a mean age of 54,8 years, while those who tested positive had a mean age of 62,3 years.

Among 59 individuals with known CeD, 10 tested positive for *H. pylori*, four had borderline results, and one lacked a serum sample.

Analysis was performed on IgG antibodies against specific *H. pylori* antigens in individuals who were *H. pylori*+ (*n* = 43) with previously unrecognised sCeD (Table 2).

These individuals with sCeD and anti-GroEL IgG antibodies were, on average, 16.4 years older at serum sampling than those without these antibodies. Similar age trends were observed with other antigens (gGT: 6 years; UreA: 9 years; HpcC: 7 years). Smaller age differences were noted for vacA and cagA. In the borderline group (data not shown), the presence of cagA antibodies mirrored GroEL’s effects. The results are presented in Table 3.

**Table 3:** Mean age of participants with previously unrecognised coeliac disease (sCeD) (*n* = 43), being *H. pylori* seropositive. Participants were further categorised by the presence or absence of IgG antibodies to various *H. pylori* antigens

*H. pylori* antigens		cagA	*n*	gGT	*n*	groEL	*n*	UreA	*n*	vacA	*n*	HcP	*n*
Mean age	Positive	61.2	17	62.3	23	67.2	26	65.2	14	60.5	2	64.3	11
	Negative	57.6	26	56.3	20	50.8	17	56.6	29	57.8	41	57.1	32

All were individuals with previously undiagnosed sCeD and carriers of *H. pylori* (see Table 2). *n* = number of individuals with either positive or negative tests

*H. pylori* infection may prompt antibody production detectable in serum [23]. IgG levels against transglutaminase-2 were, however, found comparable: *H. pylori* + group, 32.0 mg/l; and *H. pylori* − group, 32.9 mg/l.

## Comments and conclusion

Our study revealed that antigens from *H. pylori*, such as GroEL, gGT, UreA, and possibly both vacA and cagA, may affect the symptomatic expression of CeD. We believe, however, that our observations and suggestions should be seen in conjunction with the declining carriage rate of *H. pylori* in Western countries [24].

In 2013, Lebwohl and Blaser et al. [6] reported, in contrast to Crabtree et al. [16], an inverse association between the presence of *H. pylori* and CeD, which persisted even after adjusting for socioeconomic factors. They suggested that future studies should explore whether *H. pylori* modulates immune responses to gluten in individuals with CeD [6]. Since then, several studies have presented conflicting results [17–19]. In 2022, Yu et al. [20] presented a comprehensive meta-analysis, confirming an inverse correlation between CeD and the presence of *H. pylori*; however, they were unable to establish causality but referred to publications suggesting an influence of *H. pylori* on Treg lymphocytes [21–23]. The authors recommended that prospective studies should address this topic and further investigate gut microbiota and immune imbalance as potential mechanisms for disease progression in CeD [20]. *H. pylori* infection may ameliorate the severity of CeD in children [7]. In contrast, however, the aforementioned meta-analysis suggests that *H. pylori* may increase the severity of abdominal symptoms from CeD [20].

Moreover, *H. pylori* infection is characterised primarily by intense gastritis at the beginning, which then decreases in intensity and may remain stable for years. In some individuals (10–15%), it can lead to peptic ulcer disease in the duodenum or stomach [24], while in others (< 1%), it may lead to stomach cancer [11, 25]. Mucosa-associated lymphoma, i.e., MALT lymphoma in the stomach, has also been linked to *H. pylori* infection (< 0.1%) [26]. In children, the infection prompts the immune system to produce antibodies that can be detected in the serum, showing low serum IgG antibodies against transglutaminase-2 [27]. In our cohort, no such extra effect of *H. pylori* infection was observed. Reports suggest, however, that *H. pylori* may inhibit the immune response to gliadin [8].

Furthermore, there are several virulence factors in *H. pylori*. Thus, the Anti-Helicobacter-6 IgG, which is an in vitro diagnostic test for the qualitative determination of antibodies of the IgG isotype against *H. pylori*-specific antigens in serum, should catch such specific antibodies. These extracellular products contribute to pathogenesis by causing direct damage to the stomach epithelium, leading to chronic inflammation with increased levels of inflammatory agents such as interleukin 8, interleukin 1, or tumour necrosis alpha factor [10, 11, 24].

Additionally, several extraintestinal manifestations of *H. pylori* infection have been proposed and partially documented [24, 30]. The importance of autoantibodies in the development of atrophic gastritis, a precursor to stomach cancer, has been discussed [11]. A study using *H. pylori* multiplex serology [11] identified antibodies to HcpC and GroEL as independent virulence factors that, in combination with the established markers anti-CagA and anti-VacA, were highly predictive of chronic atrophic gastritis risk [11]. In a population-based cancer case-control study, antibodies to a multitude of *H. pylori* proteins were associated with stomach cancer [11]. In the present study, this technology [11–14] was applied to sera from individuals with CeD who were serologically defined as having *H. pylori* infection by the primary test (LIAISON).

Furthermore, *H. pylori* may affect the regulatory immune cells (Tregs). The Treg naïve (Tregn), which are primarily primed in the thymus, prevents attacks on self-antigens. The large group named TregE, derived from naïve CD4 T cells, is destined to identify bacterial and viral antigens as potential threats to the individual and thus to destroy them [28, 29]. The Treg response caused by *H. pylori* can change the immune response and may modulate autoimmune reactions [24]. Thus, an overview of *H. pylori* [24] summarises the recruitment of T1 cells to the human gastric mucosa, including Th1, Th17, and Treg cells. While animal models for CeD studies exist, there are no animal studies on the potential protective effects of *H. pylori* [8, 18]. Research in a mouse model, however, indicated that infection with *H. pylori* in infant mice reduces the incidence of allergies in adult mice. This effect did not occur, however, if the *H. pylori* infection was contracted in adult mice [30]. Interestingly, a potential protective effect against asthmatic disease was observed in a cohort of children, possibly linked to early-life *H. pylori* infection, as asthmatic disease was not observed at the age of 16 in children who were *H. pylori +* by the age of 2. At the age of 2 years, the prevalence of *H. pylori* carriage was approximately 6% [31]. Thus, if *H. pylori* is contracted during early childhood, the clinical symptoms of related conditions, such as CeD, may be delayed. Consequently, a reduction in *H. pylori* carriage from childhood could lead to an increase in the number of clinically overt cases of CeD.

Internationally published data indicate a global *H.pylori* carriage rate of 56.1% (ranging from 49.4% to 62.9%), while in Europe, the rate stands at 47.5% (ranging from 43.0% to 52.1%) for the corresponding age group [32]. In the current study based on the HUNT4 survey, a large number of individuals were included, revealing in contrast an average *H. pylori* carriage rate of approximately 18% [2–4]. Some of these individuals may have contracted the infection during childhood. In our subset of participants who tested positive for CeD by serology, 2.4% of those under 30 years old were *H. pylori* carriers. In contrast, 12.1% of participants over 60 years old were carriers.

This study has limitations, primarily due to the low carriage rate of *H. pylori.* As a result, there are only a few cases in each study group, leading to insufficient data for statistical analysis, which ultimately prevents us from drawing statistically significant conclusions. Moreover, PCR detection of *H. pylori* was performed, revealing fewer positive cases compared to serology. The observed trends were similar, but the number of positive cases was lower than those of *H. pylori* serology. Furthermore, complications arise when commenting on results from other studies due to the use of different methods to determine *H. pylori* status and inconsistency in controlling for age, sex, and socioeconomic status [8]. This limitation is important, as CeD may be more common in women and people with higher socioeconomic status, while *H. pylori* is more common in the opposite groups [20]. In addition, individuals infected with *H. pylori* may experience abdominal pain and other gastrointestinal issues that can mask symptoms of CeD, resulting in a delayed diagnosis. *H. pylori* infection can also influence the antigenicity of gliadin due to the effects of stomach acid [1, 8].

This study found that those who have CeD and are infected with *H. pylori*, tend to be older than similar individuals who are *H. pylori*−. As the prevalence of *H. pylori* in the community gradually decreases, a cohort effect should be considered to explain the mean age differences. Interestingly, antigens such as GroEL, gGT, UreA, and possibly both vacA and cagA from *H. pylori* or other bacteria with similar antigens [33, 34] may affect the expression of CeD, potentially delaying the clinical diagnosis of CeD if infected with *H. pylori* early in life. At present, studies have addressed the role of subsets of T cells for inflammation in CeD [35]. Furthermore, Catassi et al. [36] reported a lowered number of *Lactobacilli* and *Bifidobacterium* and an increase in *Bacteroides* and *E. coli* in individuals with CeD. However, based on evidence from a Mendelian randomised study [37], a causal relationship between *H. pylori* and CeD has not been established. Further studies are therefore warranted, comparing the microbiome of healthy individuals compared with that of individuals with CeD, while considering the hygiene hypothesis [38].

## Data Availability

Data are available upon reasonable request. Researchers with a PhD associated with Norwegian research institutes can apply for the use of HUNT material: data and samples - given approval by a Regional Committee for Medical and Health Research Ethics. Researchers from other countries are welcome to apply in cooperation with a Norwegian Principal Investigator. Access to the requested HUNT material is given after the application is approved of by HUNTs Data Access Committee (DAC) and an agreement is signed. The agreement gives the researcher(s) the right to research a specific topic for a limited time period and to publish a decided-upon number of articles. For data information contact: HUNT Research Centre, Forskningsveien 27600 Levanger, Norway. Phone +47 74 07 51 80 | +47 74 01 92 40 | +47 407 90 010. Mail: kontakt@ hunt.ntnu.no.
